# Adhesions Assemble!—Autoinhibition as a Major Regulatory Mechanism of Integrin-Mediated Adhesion

**DOI:** 10.3389/fmolb.2019.00144

**Published:** 2019-12-17

**Authors:** Rejina B. Khan, Benjamin T. Goult

**Affiliations:** School of Biosciences, University of Kent, Canterbury, United Kingdom

**Keywords:** talin, autoinhibition, integrin, vinculin, riam, cell adhesion, mechanobiology

## Abstract

The advent of cell-cell and cell-extracellular adhesion enabled cells to interact in a coherent manner, forming larger structures and giving rise to the development of tissues, organs and complex multicellular life forms. The development of such organisms required tight regulation of dynamic adhesive structures by signaling pathways that coordinate cell attachment. Integrin-mediated adhesion to the extracellular matrix provides cells with support, survival signals and context-dependent cues that enable cells to run different cellular programs. One mysterious aspect of the process is how hundreds of proteins assemble seemingly spontaneously onto the activated integrin. An emerging concept is that adhesion assembly is regulated by autoinhibition of key proteins, a highly dynamic event that is modulated by a variety of signaling events. By enabling precise control of the activation state of proteins, autoinhibition enables localization of inactive proteins and the formation of pre-complexes. In response to the correct signals, these proteins become active and interact with other proteins, ultimately leading to development of cell-matrix junctions. Autoinhibition of key components of such adhesion complexes—including core components integrin, talin, vinculin, and FAK and important peripheral regulators such as RIAM, Src, and DLC1—leads to a view that the majority of proteins involved in complex assembly might be regulated by intramolecular interactions. Autoinhibition is relieved via multiple different signals including post-translation modification and proteolysis. More recently, mechanical forces have been shown to stabilize and increase the lifetimes of active conformations, identifying autoinhibition as a means of encoding mechanosensitivity. The complexity and scope for nuanced adhesion dynamics facilitated via autoinhibition provides numerous points of regulation. In this review, we discuss what is known about this mode of regulation and how it leads to rapid and tightly controlled assembly and disassembly of cell-matrix adhesion.

## Introduction

### The Origin of Complex Cell Systems

The emergence of life is an enigmatic question that humankind has pondered for millennia. At some point millions of years ago, the last universal common ancestor (LUCA) appeared and all organisms on earth descended from this initial cell (Yutin et al., 2008), or so the story goes. The steps to produce such an organism are controversial and mystifying. However, evolution from this single cell seems somewhat easier to imagine. A key step in the formation of multicellularity and more complex organisms was the development of cell adhesion molecules. The ancient origins of integrin-mediated adhesions has been traced back to the genesis of multicellularity (Sebé-Pedrós et al., 2010; Brunet and King, 2017).

Initially, these cell adhesion molecules enabled cells to form interactions with other cells to form cell-cell junctions and, in animal cells, interactions with the newly-acquired extracellular matrix (ECM) to form cell-matrix junctions. The ability of cells to form sheets of cells and attach to an underlying matrix was central to the development of multicellular animal life.

These attachment points also developed into sensitive sensory modules, able to feel the mechanics of the microenvironment and adopt the role of mechanotransduction centers—enabling cells to monitor and respond to mechanical cues and convert them into biological signals to elicit different cellular responses.

### Autoinhibition as a Regulator of Protein Activity

**Figure 1:**
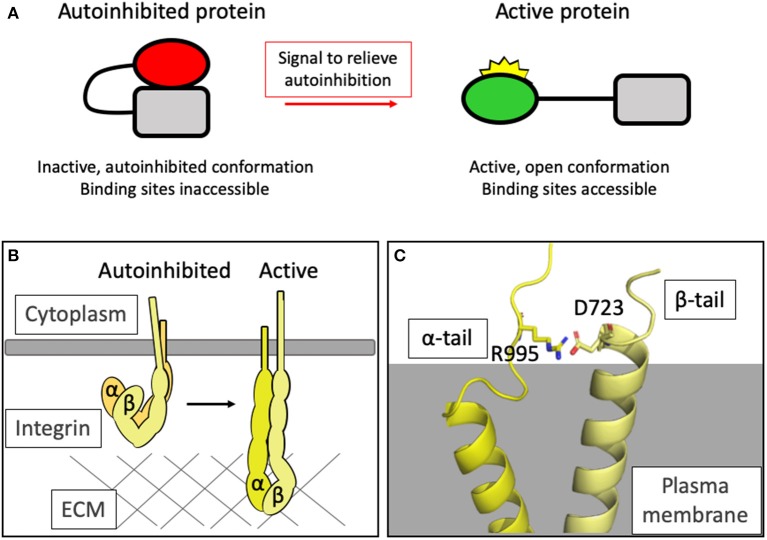
Autoinhibition as a means of regulating protein activity. **(A)** Simplified schematic of the general principle of autoinhibition. **(B)** Integrins exist in a closed, autoinhibited conformation when inactive (left). Upon activation, the headpiece extends and the cytoplasmic tails are held further apart. In this high-affinity conformation, the headpieces can interact with the extracellular matrix and an adhesion can assemble. **(C)** The structure of the complex formed between the transmembrane domains of integrin αIIb and β3 [PDB ID: 2K9J Lau et al., 2009] showing the salt bridge formed between R995 of αIIb and D723 of β3 which maintains the integrin in the autoinhibited state.

Key to the development of complexity is the ability to regulate adhesion and control the proteins that assemble together to form adhesive structures. One way that proteins can be dynamically regulated is via formation of an intramolecular interaction that maintains the protein in an inactive state until adhesion assembly is required. The regulation of autoinhibition, and the factors that enable regulation of the activity of the protein, provides regulatable checkpoints in the system (Figure 1A).

The concept of autoinhibitory domains can be found throughout biology—these are regions of proteins which can form intramolecular interactions which regulate behavior and activity. We refer the reader to the review on autoinhibitory domains by Pufall and Graves as it provides an excellent, still highly relevant description of the fundamentals of autoinhibition (Pufall and Graves, 2002).

## Cell-matrix Adhesions: Attachment and Signaling Centers

### Integrin Adhesion Complexes

The affinity of integrins for the matrix is regulated by the process of integrin activation whereby the integrin that resides in a low-affinity, compact arrangement at rest is converted to, and stabilized in, an extended high-affinity conformation for ligand (Shattil et al., 2010; Sun et al., 2016). Integrin activation serves as a paradigm for how protein activity can be regulated by conformation (Figure 1B). Following activation, huge protein complexes coalesce onto the short cytoplasmic integrin tails, ultimately giving rise to large signaling complexes. The order of this assembly is the focus of intense research.

### Autoinhibition at the Heart of Integrin Adhesion Complexes

Many of the proteins that assemble to form integrin adhesion complexes are recruited to the adhesion site in an inactive state and, upon receiving the correct signals, are activated and set to work mediating adhesion and mechanotransduction. As we will see later, autoinhibition is not limited to the pre-adhesion phase of the protein's life; autoinhibition plays a central role in coordinating the dynamic assembly and disassembly required for cell migration—cells need to adhere and detach in perfect synchrony to enable progressive directional movement. Due to limitation in space, it is not possible to detail all of the adhesion proteins that are regulated by autoinhibition. Here, we will focus on some of the best characterized to serve as paradigms of autoinhibition regulating adhesion.

### Integrins

Perhaps the best place to start is at the integrins themselves. The inside-out/outside-in activation of integrins represents a textbook example of autoinhibition and the regulation of ligand-binding affinity via allosteric effectors.

Whilst the concept of adhesions being formed between cells and the substratum had been appreciated since the work of Abercrombie (Abercrombie, 1961), integrins, as the receptors mediating these cell-ECM links, were first characterized in the 80s (Tamkun et al., 1986) and over the last 35 years have been the subject of extensive research effort (a PubMed search for the term “integrin” on 9th September 2019 yields 73,512 results). The integrin family of proteins consists of transmembrane, α/β-heterodimeric cell surface adhesion receptors that generally consist of a large ectodomain, a single transmembrane domain and a short cytoplasmic tail domain. This structure facilitates the characteristic bidirectional signaling ability of integrins wherein signals that regulate countless crucial cellular activities can be transmitted across the plasma membrane. In vertebrates, 18 α- and 8 β-subunits have been identified which form non-covalent links in different combinations to generate 24 integrin heterodimers (Hynes, 1992). Many great reviews have covered the complexity of bidirectional signaling through integrins (Qin et al., 2004; Luo and Springer, 2006; Shattil et al., 2010; Campbell and Humphries, 2011) but it is worth a quick summary here.

The concept of integrin activation has been around for over 30 years, and the first paper showing that conformational changes regulate integrins was published in 1990 (Frelinger et al., 1990). The concept of “outside-in” signaling, whereby extracellular ligands can trigger large conformational changes to the integrin structure leading to increased integrin activation, quickly followed (Du et al., 1991). The use of monoclonal antibodies that recognized epitopes on the integrins that are only accessible in certain conformations led to the notion of allosteric behavior of integrins (Mould et al., 1996; Askari et al., 2009) mediated by autoinhibition.

“Inside-out” signaling refers to integrins sensing intracellular signals, resulting in the binding of talin and kindlin to the cytoplasmic tail of the β-subunit leading to activation of the integrin. In reality, it is likely that both of these bidirectional signaling axes work in tandem, with extra- and intracellular cues contributing constantly to orchestrate the overall dynamics of the integrin.

The structural changes in integrin conformation that occur upon activation, leading to the relief of autoinhibition and exposure of the ligand binding sites both extracellularly and intracellularly, are extensive and result in large-scale reorganization of the ectodomains (Ye et al., 2012) and separation of the transmembrane and cytoplasmic tail domains (Kim, 2003) (Figure 1B).

Integrins are generally thought to have three major conformations: a bent, closed form, an extended form with the headpiece of the ectodomain still closed, and an extended form with the headpiece open and the α- and β-cytoplasmic tails a greater distance apart (Shimaoka et al., 2002; Li et al., 2017). The binding sites for many integrin ligands are cryptic—integrin activation causes a switch to the extended, open conformation and triggers exposure of these surfaces.

Autoinhibition in the intracellular region is maintained via an electrostatic interaction between the two cytoplasmic tails: for example, in αIIbβ3 integrin, Asp723 in the β3 tail binds Arg995 in the αIIbβ3 tail (Anthis and Campbell, 2011) (Figure 1C). This salt bridge is crucial for maintaining the low-affinity state, holding the legs together (Hughes et al., 1996; Vinogradova et al., 2002; Kim et al., 2009; Lau et al., 2009) and preventing the separation of the legs that drives activation of the integrin.

Protein-protein interactions between the β-tail and the actin-binding protein talin (see next section) can relieve this autoinhibitory interaction and drive tail separation, propagating a conformational rearrangement of the ectodomains and unfurling to reveal the ligand-binding motifs (Harburger and Calderwood, 2009). The structure of the talin2 F2F3 domains bound to the cytoplasmic tail of the β1d tail [PDB ID: 3G9W Anthis et al., 2009] revealed that part of the activation process mediated by the talin head binding to integrins (Calderwood et al., 1999) was to not just break this salt bridge but to form an alternate salt bridge between the Asp723 and a conserved basic residue in talin [Lys327 in mouse talin2 Anthis et al., 2009]. Active talin, with a little help from the FERM domain protein kindlin (Rogalski et al., 2000; Moser et al., 2008), can therefore convert integrin from an autoinhibited to an active conformation.

### Talin

**Figure 2:**
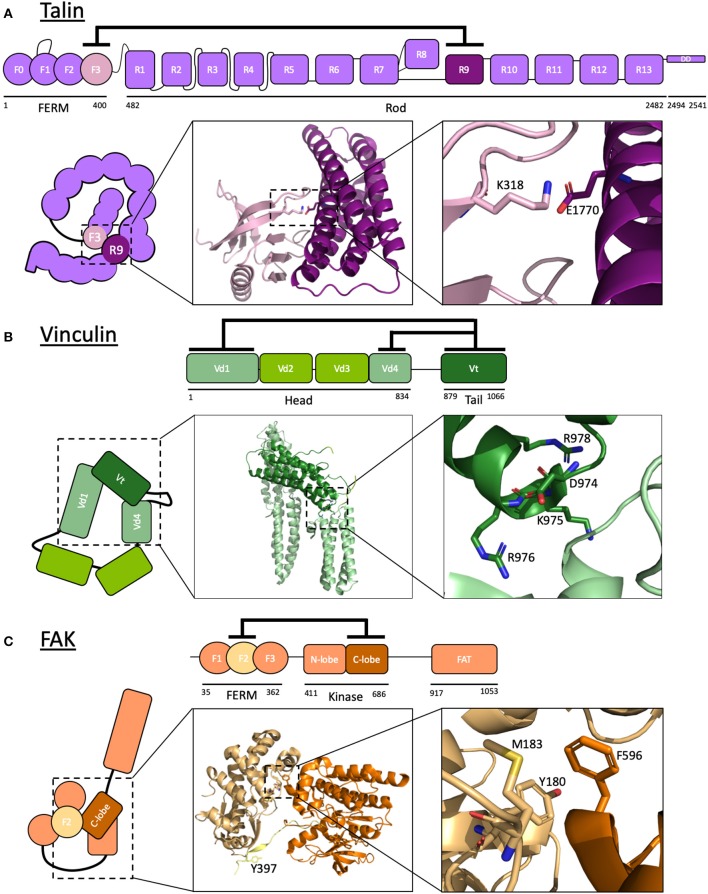
Autoinhibition of focal adhesion proteins. **(A–C)** Schematic diagrams of active and autoinhibited **(A)** talin, **(B)** vinculin, and **(C)** FAK. Insets: The crystal structures of the autoinhibitory interactions are shown. **(A)** Talin: F3 of the FERM domain and R9 of the rod are the primary interacting domains in talin autoinhibition. K318 and E1770 are required for this interaction to take place, forming a key buried salt bridge. Adapted from crystal structure PDB ID: 4F7G (Song et al., 2012). **(B)** Vinculin: the vinculin tail, Vt, interacts with both Vd1 and Vd4 domains of the head to form a strong autoinhibitory conformation. Mutating residues D974, K975, R976, R978 (Cohen et al., 2005) results in a constitutively active vinculin by disrupting the interaction between Vt and Vd4. Adapted from crystal structure PDB ID: 1TR2 (Borgon et al., 2004). **(C)** Focal adhesion kinase: F2 of the FERM domain of FAK interacts with the C-lobe of the kinase domain to keep FAK in a closed conformation, rendering a key tyrosine in the linker between these domains (Y397) inaccessible to phosphorylation. Adapted from crystal structure PDB ID: 2J0J (Lietha et al., 2007). Images made using PyMOL.

Talins are ~270 kDa adaptor proteins involved in integrin-mediated adhesions and were first discovered in adhesion plaques (cell-ECM junctions) in fibroblasts (Burridge and Connell, 1983). The two isoforms, talin1 and talin2, are encoded by separate genes (Senetar and McCann, 2005) but have the same domain structure consisting of a non-canonical linear FERM domain (F0-F3) connected, via a linker, to a rod domain comprised of 13 α-helical bundles (R1-R13) followed by a C-terminal dimerization domain (DD) (Calderwood et al., 2013; Goult et al., 2013b) (Figure 2A).

Talin activates integrins by binding to the cytoplasmic tail of the β-integrin subunit via a phosphotyrosine-binding (PTB)-like region in F3—this domain is termed integrin binding site 1 (IBS1). As mentioned in the previous section, the interaction between the talin head domain with integrin is sufficient to relieve autoinhibition of the integrin, exposing the previously cryptic binding sites for integrin ligands. However, talin itself is regulated by multiple layers of autoinhibition.

The first evidence that talin was autoinhibited came from the initial structural and biochemical characterization of talin in 1987 (Molony et al., 1987). Electron microscopy analysis of purified talin from chicken gizzard smooth muscle revealed that talin was a flexible elongated molecule but could adopt a more globular compact form at low ionic strength. It has since been demonstrated that the autoinhibition of talin into this compact cytosolic conformation (Goult et al., 2013a; Dedden et al., 2019) occurs primarily via an interaction between the integrin binding site in F3 and the rod domain R9 (Goksoy et al., 2008; Goult et al., 2009; Song et al., 2012) with additional weaker interactions including that between F2F3 and R1R2 (Banno et al., 2012; Goult et al., 2013a). The compact autoinhibited structure (Molony et al., 1987; Goult et al., 2013a) is facilitated by the formation of a talin homodimer, formed via the dimerization domain (DD) at the very C-terminal helix (Gingras et al., 2008). This stabilizes the autoinhibited conformation via the various inter- and intramolecular interactions taking place. In this configuration, the integrin binding site in the F3 domain and the actin-binding sites in the rod are masked which implies that, in order for integrin activation and subsequent signaling to occur, a conformational change must be induced in order to relieve talin autoinhibition.

#### Layers of Talin Autoinhibition

Many binding sites in talin are concealed as a result of these layers of autoinhibition (Gough and Goult, 2018)—most notably the sites for integrin, actin and vinculin—but also the sites in the rod with which the majority of talin binding partners interact. The folded conformation has the rod domains wrapped around the edge of the compact conformation with the two head domains of the dimer buried inside (Goult et al., 2013a). The rod domains have binding sites on their faces, some of which will be buried inside the closed conformation and inaccessible. However, some binding sites are still accessible—for example, Rap1-interacting adapter molecule (RIAM) is able to interact with autoinhibited talin, binding to a folded surface on R2R3 which is outward-facing. RIAM-mediated coupling of talin to Rap1's membrane-targeting motifs is a key event before integrin activation can occur (Lee et al., 2009, 2013; Shattil et al., 2010; Yao et al., 2014a; Lagarrigue et al., 2015).

R9 and integrin bind to the same site on F3, indicating that the autoinhibitory interaction of talin and its integrin binding are mutually exclusive. The specific sites for talin autoinhibition have been investigated: on R9, these include a negatively charged surface comprised of residues Asp1676, Asp1763, Glu1770, Glu1798, and Glu1805 (Goult et al., 2009) which bind to the positively charged integrin “activation” loop on F3 including Lys316, Lys318, Lys320, Lys322, and Lys324 (Goksoy et al., 2008; Goult et al., 2009; Song et al., 2012). The crystal structure of the complex between F2F3 and R9 [PDB ID: 4F7G Song et al., 2012] and the recent cryo-EM structure of the autoinhibited form of talin (Dedden et al., 2019) have provided atomic detail of this interface and confirmed the key role of Glu1770 in mediating autoinhibition via a buried salt bridge with Lys318. Mutations to Glu1770 in R9 have been shown to reduce the autoinhibitory interaction (Goult et al., 2009), and this mutant has allowed detailed analysis of talin uncoupled from the upstream signaling pathways (see later section “Manipulation of autoinhibition by mutation”).

The layers of autoinhibition in talin extend beyond the head-tail interaction. Once this “top layer” of autoinhibition is relieved, the binding sites on talin for integrin and actin are exposed and form the core of the adhesion complex. Talin has three actin-binding sites (Hemmings et al., 1996) and the regulation by autoinhibition of two actin-binding sites in the rod region, ABS2 and ABS3, demonstrates two further layers of talin regulation. The C-terminal ABS3 (McCann and Craig, 1997; Gingras et al., 2008) is comprised of the two R13 domains of the dimer linked together via the DD. ABS3 is inaccessible in the closed, compact form (Goult et al., 2013a) but, upon talin unfurling, is available to bind actin. The affinity of ABS3 for actin is further regulated via autoinhibition *within* the R13 domain, as the first helix (upstream helix, USH) of R13 limits actin binding (McCann and Craig, 1997). This domain-level autoinhibition requires force and/or changes in local pH (Srivastava et al., 2008) to allow maximal actin binding. Once ABS3 engages actin, it can capture the retrograde flow of actin that begins to exert forces onto the tethered talin molecule. These forces can cause conformational changes in talin and relieve autoinhibition or disrupt binding interactions of other domains. The second actin binding site in talin, ABS2, is in domains R4–R8 in the middle of the talin rod (Atherton et al., 2015). In the absence of force, ABS2 is cryptic and maintained in a low-affinity autoinhibited state via the adjacent domains R3 and R9 (Atherton et al., 2015). Forces exerted on talin via ABS3 relieve this autoinhibition and reveal ABS2 which can then form the high-affinity, tension-bearing cytoskeletal linkages with actin (Atherton et al., 2015; Kumar et al., 2016; Ringer et al., 2017). Here, mechanical force is the major driver relieving ABS2 autoinhibition, but this only occurs following relief of the layers of autoinhibition preceding it: i.e., talin head-tail autoinhibition and the changes in ABS3 that facilitate actin binding to talin.

Many of the binding partners of talin require sites which are not constitutively accessible. Rod domain partners may bind when domains are folded, unfolded, or at intermediate levels of folding (Goult et al., 2013b, 2018). These sites can be exposed by various stimuli; for example, talin has 11 cryptic vinculin-binding sites (VBS) which are buried in the hydrophobic core of the folded rod helical bundles. These VBS are exposed when talin is under force, causing sequential rod domain unfolding and allowing the first subdomain of the vinculin head, Vd1, to bind (Hytönen and Vogel, 2008; del Rio et al., 2009; Yao et al., 2016). Vinculin binding to these VBS subsequently allows for stabilization and maturation of the adhesion (Yao et al., 2014a). Each VBS-containing rod domain has a different force threshold at which it unfolds, leading to different forces required to relieve autoinhibition of each VBS. Further, each VBS helix has a different mechanical stability, and so the VBS-vinculin interactions at each site also have different strengths (Wang et al., 2019).

It remains to be determined exactly which binding sites are available in each conformation, and the catalog of talin ligands is expanding constantly (Goult et al., 2018). Furthermore, the lifetimes of each conformation are regulated by autoinhibition modulated by many different signaling cues including PTMs, force and calpain cleavage (see later section “Control of autoinhibition”).

This leads to a domino effect of autoinhibitory relief steps downstream of Rap1 activation: (i) RIAM is activated, which translocates talin to the plasma membrane where (ii) the talin head-tail autoinhibition is relieved. (iii) Once active, talin relieves the autoinhibition of integrin. (iv) By connecting integrins to the actin retrograde flow, talin autoinhibition is further relieved, first with activation of enhanced actin binding to ABS3. (v) As force increases, mechanical activation of vinculin-binding sites in talin occurs, initially by unfolding of the R3 domain, the least stable of the rod domains (Goult et al., 2013b; Yao et al., 2014a). (vi) Mechanical exposure of high-affinity actin binding takes place via relief of autoinhibition of ABS2. This is just one example of a simplified, linear route through the autoinhibitory landscape regulating adhesion assembly; many other factors can feed into and modulate these steps and the order in which they occur.

### Vinculin

Like talin, vinculin was first discovered as a component of adhesion plaques (Geiger, 1979). Vinculin is a ~116 kDa actin-binding protein comprised of a large, globular head consisting of four α-helix-containing domains (Vd1–Vd4) linked to a tail domain (Vt) by a proline-rich hinge region (Figure 2B).

Vinculin interacts with various proteins involved in integrin-mediated cell-ECM adhesion and cadherin-mediated cell-cell adhesion including α-actinin, vinexin, and ARP2/3 (Wachsstock et al., 1987; Kioka et al., 1999; DeMali et al., 2002). However, its most notable binding partners are talin and filamentous actin (Burridge and Mangeat, 1984). Vinculin also has a similar function at cell-cell adhesions, stabilizing the junctions via interaction between actin and α-catenin (Bays and DeMali, 2017). This linkage is crucial for mechanotransduction (Huveneers and de Rooij, 2013; Zaidel-Bar, 2013; Yao et al., 2014b) and stabilization of cadherin at the cell surface (Peng et al., 2010).

When talin is subjected to force, sequential unfolding of helical bundles of the rod domain occurs which exposes up to 11 cryptic vinculin binding sites (VBS) to which Vd1 can bind (Izard et al., 2004; Papagrigoriou et al., 2004). Filamentous actin, on the other hand, interacts with Vt (Johnson and Craig, 1995).

Autoinhibition of vinculin, like talin, is mediated by a head-tail interaction that conceals the binding surfaces for most known ligands, including for talin and actin (Cohen et al., 2005, 2006). This interaction occurs via two interfaces: between Vd1 and Vt and between Vd4 and Vt. Although these are individually low-affinity, the combined interaction amounts to a strong autoinhibitory interaction (Cohen et al., 2005). The crystal structure of full-length vinculin in its autoinhibited form (Bakolitsa et al., 2004; Borgon et al., 2004) shows the two head domains interacting with Vt. A constitutively active vinculin termed vinculin T12 has been developed wherein a cluster of four charged residues in Vt are mutated: Asp974Ala, Lys975Ala, Arg976Ala, and Arg978Ala. This mutant has significant loss of affinity to the vinculin head compared to wildtype vinculin (Cohen et al., 2005). More recently, an improved constitutively active vinculin has been developed, the T12K mutant, with Asp974 mutated to a lysine (Asp974Lys) to further destabilize the interaction with the head (Chorev et al., 2018). Constitutively active vinculin markedly reduces adhesion turnover (Humphries et al., 2007; Carisey et al., 2013), and locks talin into an extended conformation (Yao et al., 2014a). Not surprisingly, this loss of adhesion dynamics is lethal in flies (Maartens et al., 2016).

### Focal Adhesion Kinase (FAK)

The mechanisms underlying autoinhibition of focal adhesion kinase (FAK) are well-characterized and, being a kinase, a striking example of coupling regulation of protein activity to downstream signaling cascades. FAK is another linear multidomain protein with numerous binding sites for ligands. As the name suggests, FAK contains an enzymatically active kinase domain able to phosphorylate tyrosine residues in target proteins. FAK comprises an N-terminal FERM domain, a kinase domain, a ligand-binding region and a C-terminal 4-helix bundle termed the focal adhesion targeting (FAT) domain (Figure 2C).

At rest, FAK adopts an autoinhibited, closed conformation where the FERM domain directly binds and occludes both the catalytic cleft and the activation loop of the kinase domain (Cooper et al., 2003; Lietha et al., 2007). The crystal structure of autoinhibited FAK allowed identification of the residues on each domain involved in the interaction (Lietha et al., 2007). In this closed conformation, the FAK activation loop is sequestered, preventing autophosphorylation of Tyr397. Interestingly, FERM-mediated autoinhibition of kinase activity has also been observed in the Janus Kinase (JAK) family. JAK proteins have a similar domain structure to FAK and share this common mode of regulation (Zhou et al., 2001).

Two mutants have been designed which significantly increase kinase activity of FAK: a double mutant in F2 of the FERM domain (Tyr180Ala and Met183Ala) and a mutation in the kinase domain (Phe596Asp) (Lietha et al., 2007).

Activation of FAK requires relief of autoinhibition and this can occur through the FAK-FERM domain engaging the integrin tail, leading to release of the activation loop and autophosphorylation. As a result, binding between FAK and the Src tyrosine kinase is enhanced, leading to an active FAK-Src kinase complex able to activate and regulate many other proteins as a major driver of adhesion signaling (Schlaepfer et al., 1999; Zhao and Guan, 2011; Horton et al., 2016).

Further, upon release of autoinhibition, FAK can become part of a tethered linkage in force-transmission pathways, tethering to integrins via its FERM domain while the C-terminal region couples to cytoskeletal proteins. This suggests that mechanical force may also contribute to the lifetime of the active form of the protein (see later section: Mechanical regulation of autoinhibition).

### Rap1-Interacting Adapter Molecule (RIAM)

**Figure 3:**
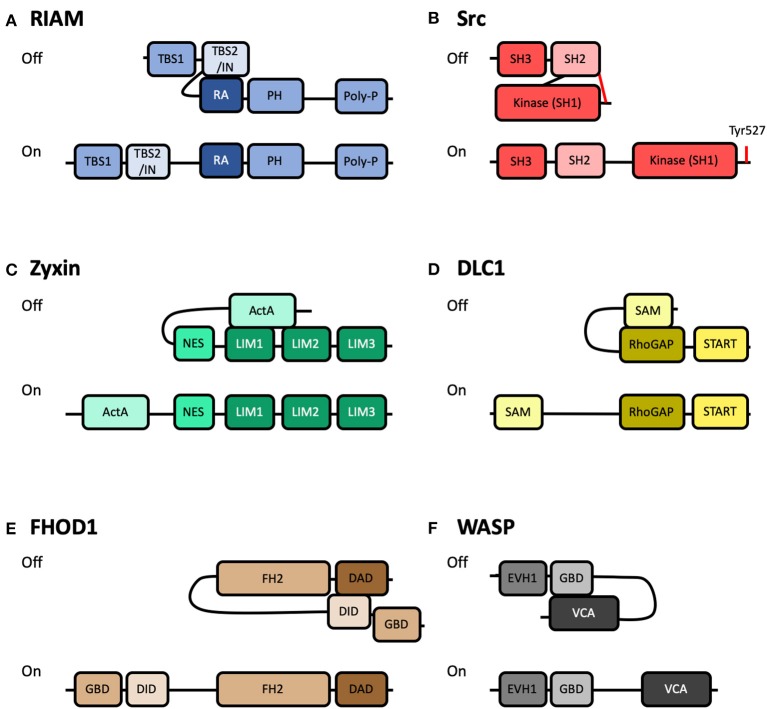
Schematics of autoinhibited and activated forms of other integrin-based adhesion-associated proteins. Cartoon representation of some of the known adhesion proteins that are regulated by autoinhibition. Top: Autoinhibited (Off) conformation; Bottom: Active (On) conformation. **(A)** RIAM, comprised of two Talin-Binding Sites (TBS), and an INhibition motif (IN), a Ras-Association domain (RA), a Pleckstrin Homology (PH), and a Poly-proline motif. Autoinhibition is mediated by interaction between the IN and RA domains (Chang et al., 2019). **(B)** Src, comprised of a Kinase domain (SH1), a Src homology 2 domain (SH2) and a Src homology 3 domain (SH3). Autoinhibition is mediated by interaction between the SH2 domain and phosphorylated Tyr527 at the C-terminus (Shenoy et al., 1992). **(C)** Zyxin, comprised of an N-terminal region with Proline-rich “ActA” repeats, Nuclear Export Sequences (NES), and three LIM domains. Autoinhibition is mediated by interaction between the ActA and LIM domains (Nix et al., 2001; Call et al., 2011). **(D)** DLC1, comprised of a Sterile Alpha Motif (SAM), a Rho-GTPase Activating Protein domain (RhoGAP) and a STeroidogenic Acute Regulatory protein-related lipid-Transfer domain (START). Autoinhibition is mediated by interaction between the SAM and RhoGAP domains (Kim et al., 2008). **(E)** FHOD1, comprised of a GTPase-Binding Domain (GBD), a DAD Interacting Domain (DID), a Formin-Homology 2 (FH2) and a Diaphanous-Autoregulatory Domain (DAD). Autoinhibition is mediated by interaction between the DID and DAD domains (Takeya et al., 2008). **(F)** WASP, comprised of an Ena/VASP Homology-1 domain (EVH1), a GTPase-Binding Domain (GBD), and a Verprolin homology, Central hydrophobic and Acidic domain (VCA). Autoinhibition is mediated by interaction between the GBD and VCA domains (Kim et al., 2000).

An important regulator of the integrin adhesion complex assembly is Rap1-interacting adapter molecule (RIAM). RIAM is a Rap1 effector that can interact directly with talin and translocate it to the plasma membrane, and thus into close proximity to the integrins (Han et al., 2006; Lee et al., 2013). The direct interaction of RIAM and talin is mediated via the N-terminus of RIAM which contains two talin binding sites (TBS) which interact with four of the talin rod domains (Goult et al., 2013b), with high-affinity binding occurring to the R2 and R3 talin rod domains. RIAM is another linear molecule, comprised of the two TBS and a long flexible linker connecting to Ras association (RA) and pleckstrin homology (PH) domains (Figure 3A).

The structural basis of RIAM autoinhibition was recently shown to be mediated by the region between the two TBS and overlapping with TBS2, termed the inhibitory region (IN) (Figure 3A). This IN region, mapped to residues 27–93, was shown to interact with, and occlude, the Rap1 binding site on the RA domain (Zhang et al., 2014; Chang et al., 2019) via a switchblade-type autoinhibitory interaction. A tyrosine residue forming part of the binding site, Tyr45, was also shown to be a substrate for focal adhesion kinase (FAK). This suggests a novel regulatory axis whereby FAK-mediated phosphorylation of RIAM relieves RIAM autoinhibition, exposing both the RA and TBS domains, increasing the ability of RIAM to (i) co-localize with Rap1 at the leading edge of cells and (ii) to recruit talin to the same location. A Glu60Ala/Asp63Ala mutation was shown to render RIAM constitutively active (Chang et al., 2019).

This Rap1:RIAM:talin nexus is thus tightly controlled in a myriad of ways, and the dynamic balance of activity status of each protein is implicitly entangled. RIAM is likely activated by active Rap1 (Bos, 2005; Stefanini and Bergmeier, 2016), and the interaction with talin is likely to tilt the equilibrium balance further. The interaction between the PH domain and membrane phospholipids will further tip the equilibrium. The lifetime of the interaction can then be controlled by phosphorylation via FAK and dephosphorylation by the relevant phosphatase. All of these factors will control the activation state of RIAM, highlighting the complex balance of factors that determine the lifetime of interactions.

### Src

Relief of autoinhibition of FAK following activation at adhesion sites leads to FAK autophosphorylation of Tyr397 (Frisch, 1996) which provides one mechanism for the recruitment of Src to adhesion complexes. Src, like FAK, is a non-receptor tyrosine kinase that associates with sites of adhesion. The cellular Src protein was originally discovered from its homology to the Rous sarcoma virus oncogene protein product, v-Src (Bishop et al., 1978). Determining the domain structure of Src transformed the field of cell signaling as it is comprised of three modular domains: a kinase domain (Src Homology 1), a phosphotyrosine-binding SH2 domain (Src Homology 2) and a polyproline-recognizing SH3 domain (Src Homology 3). Identification of these modular binding domains in Src (Pawson and Gish, 1992) set a paradigm for cell signaling pathways. Interestingly, Src is maintained in an inactive cytosolic autoinhibited state as the C-terminus of Src contains a tyrosine, Tyr527, which when phosphorylated interacts with Src's own SH2 domain (Shenoy et al., 1992) (Figure 3B). Interestingly, v-Src is constitutively active as it lacks this regulatory C-terminal autoinhibition motif. Phosphorylation of FAK Tyr397 can also enhance Src activation since the Src SH2 domain binds FAK, thus displacing the autoinhibitory tail. Relief of Src autoinhibition can be sustained by phosphatases that dephosphorylate Tyr527 and switch the molecule to its active form.

Src recruitment to, and phosphorylation of, downstream molecules allows many pathways to be regulated through Src, and drives crosstalk between integrin, Src and Rho-family GTPases (Huveneers and Danen, 2009). This provides one route through the complex activation process where a sequence of autoinhibitory interactions are relieved: integrin activation, leading to increased FAK activation, which leads to increased Src activation. All of these enhanced activities can be further modulated by post-translational modification.

### And the Rest…

Many other adhesome components have also been shown to autoinhibit. The core adhesion proteins are all long linear molecules so it is possible that they all contain autoinhibitory domains. Table 1 contains a non-exhaustive list of adhesion proteins regulated in this way, and constitutively activating mutations that can be introduced.

**Table 1:** Adhesion proteins regulated by autoinhibition.

**Protein**	**Primary interacting regions**	**Constitutively active mutant**	**References**
Integrin	Electrostatic interaction between the two cytoplasmic tails	R995A in αIIb or D723A in β3	Hughes et al., 1996
Talin	F3 and R9	E1770A	Goult et al., 2009
Vinculin	Vd1 and Vt/Vd4 and Vt	“T12”: D974A, K975A, R976A, R978A	Cohen et al., 2005
		“T12K”: D974K, K975A, R976A, R978A	Chorev et al., 2018
RIAM	IN and RA	E60A, D63A	Chang et al., 2019
FAK	F2 and kinase C-lobe	Y397F	Lietha et al., 2007
Src	C-terminus and SH2	Y527F	Shenoy et al., 1992
DLC1	SAM and RhoGAP domains	delta SAM	Kim et al., 2008
α-actinin	CaM-like and neck-R1 domains	NEECK mutant	Young, 2000; Ribeiro et al., 2014
Filamin A	Interactions in its immunoglobulin repeats, inc. I20 and I21	I2144E	Lad et al., 2007
FHOD1	Diaphanous Inhibitory Domain (DID) and Diaphanous Autoregulatory Domain (DAD)	S1131D, S1137D, T1141D	Takeya et al., 2008
WASP	GBD and VCA domains	delta C	Kim et al., 2000
Zyxin	ActA and LIM region	S142D	Nix et al., 2001; Call et al., 2011

The autoinhibited and active conformations of the proteins zyxin, WASP, FHOD1, and DLC1 are shown in Figure 3. These are just a small selection of the 200+ proteins associated with integrin adhesion complexes, but highlights the similarities between the regulatory mechanisms. In each case, the activity of the protein is controlled by a head-tail interaction which is mediated via an autoinhibitory domain binding to, and occluding, a major binding site. Very few proteins in cells are constitutively active.

## Control of Autoinhibition

Studies into the regulatory complexity of proteins' activation status is revealing adhesions to be highly dynamic. With recent advances in microscopy, it is now possible to observe constant switching between inactive and active conformations of proteins within an adhesion, as exemplified by the mounting evidence that integrins segregate into distinct nanoclusters within focal adhesions (Rossier et al., 2012; Spiess et al., 2018; Orré et al., 2019). Mutants that perturb talin autoinhibition [E1770A Ellis et al., 2013; Haage et al., 2018] or vinculin autoinhibition [T12 Cohen et al., 2005; Carisey et al., 2013] also result in strong attenuation of adhesion dynamics. Together, these results suggest autoinhibition of adhesion components is critical to the tight, dynamic regulation.

### Methods of Relief of Autoinhibition

**Figure 4:**
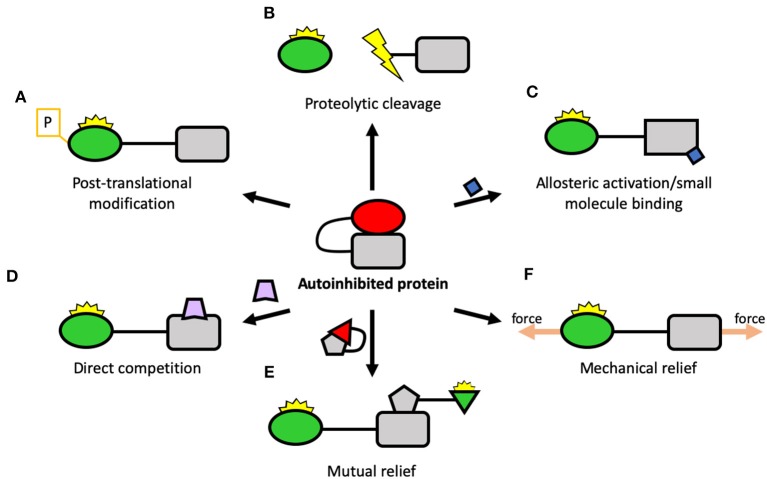
Potential ways of regulating autoinhibition. Center: A schematic of a protein regulated by autoinhibition, where an autoinhibitory domain (square) binds to, and inhibits, a ligand-binding domain (circle). There are many ways that autoinhibition can be relieved including: **(A)** Post-translational modification: a reversible way to disrupt the autoinhibitory interaction and activate the protein; **(B)** Proteolytic cleavage: by severing the link between the two domains, the protein can be constitutively activated; **(C)** Allosteric activation: another protein, small molecule, lipid surface, etc. binds to the protein and triggers conformational change which leads to activation; **(D)** Direct competition: a protein interacting with the target protein opens up the molecule; **(E)** Mutual relief: two autoinhibited proteins come together to relieve each other's autoinhibition, leading to simultaneous activation; **(F)** Mechanical relief: the two domains form part of a mechanical linkage where, by being tethered apart, they are maintained in an active conformation.

There are numerous ways that autoinhibition of proteins is controlled with multiple entry points for the regulation of protein activity, some of which are shown in Figure 4. The equilibrium between autoinhibited and active protein is exquisitely poised, such that a subtle shift toward a more active state of one protein can be sufficient to perturb the system and cause large-scale changes to cellular processes. Rather like the “butterfly effect” in chaos theory where a minor change in the initial conditions can lead to large changes in the system overall (Lorenz, 1962), these chaotic events give rise to order in the cellular world. Upregulation of a protein in response to a signal of some kind, say activation of a GTPase or interaction of two proteins, can trigger rapid amplification and cascades of activation steps that result in large global changes in the adhesion and the cytoskeleton of a cell.

One common mechanism for relieving autoinhibition is **post-translational modifications** (PTMs) (Figure 4A). Here, a kinase or other enzyme chemically alters the target protein and relieves the autoinhibition, switching the protein “on” (or “off” in the case of proteins like Src). PTMs can also stabilize an active state once open and thus control the lifetime of the active state. The most well-studied PTM is phosphorylation but there are many others, including acetylation, methylation (Gunawan et al., 2015), sumoylation (Huang et al., 2018), etc. The converse of this is the removal of PTMs, where a second enzyme such as a phosphatase removes the PTM and reverses the switch.

As well as temporary PTMs, there are also irreversible modifications that can occur, such as **proteolytic cleavage** (Figure 4B). Here, the protein is cleaved, separating into two polypeptides. The best-characterized proteases linked to adhesion belong to the calpain family of calcium-dependent cysteine proteases which cleave many adhesion components. Talin has been shown to be a substrate for calpain (Franco et al., 2004), as have paxillin and FAK (Chan et al., 2010). Calpain cleaves talin in three sites: one site in the neck liberating the head from the rod (Franco et al., 2004), one site at the C-terminus immediately before the dimerization domain (Bate et al., 2012), and a cryptic third site in the R10 domain (Zhang et al., 2012). Each of these cleavage sites will result in constitutively active talin fragments. Interestingly, whilst the initially-identified role of calpain cleavage was primarily to regulate adhesion disassembly (Franco et al., 2004), recent work has shown that calpain cleavage activation of talin is required at adhesion genesis to trigger proper adhesion formation, recruitment of further non-cleaved talin and adhesion maturation (Saxena et al., 2017), with the cleavage products playing key additional, presumably non-adhesive roles.

Many proteins are activated by interaction with an activating molecule—this can be a protein, the membrane, etc. Here, the activating molecule can activate the target via **allosteric activation** (Figure 4C) where binding causes conformational changes to the target leading to relief of autoinhibition. This can also be via a **small molecule binding** (Figure 4C): for instance, calpain proteases are activated by influx of calcium and, once active, can activate other proteins by cleavage. Another scenario is that binding of the activating moiety can activate by **direct competition** (Figure 4D) with the autoinhibitory interaction. For instance, talin can be activated by the plasma membrane, where the interaction between the plasma membrane and the F3 domain of talin is stronger than the autoinhibitory interaction holding talin in a closed conformation (Saltel et al., 2009; Song et al., 2012), causing talin to undergo large-scale reorganization upon membrane engagement.

A further scenario that is likely to be playing a major role in adhesion assembly is the **mutual relief of autoinhibition** (Figure 4E). Here, two proteins in an “off” conformation come together and mutually activate each other. Alternatively, one protein becomes activated and, in doing so, is able to activate another, triggering a “domino rally”-style activation cascade.

### Mechanical Relief—Mechanotransduction

A common theme of adhesion regulation by autoinhibition is that the proteins are regulated by head-tail autoinhibition. Here, the protein folds in a manner that is mediated by autoinhibitory domains which, when interacting, mask binding sites for ligands. Upon relief of autoinhibition, the protein unfurls to reveal the active form and expose additional binding sites. The protein changes dramatically from a more compact form to an open and extended conformation. The protein will be in equilibrium between the closed and the open states, and the lifetime of each state will be controlled by the factors described in the previous section.

**Figure 5:**
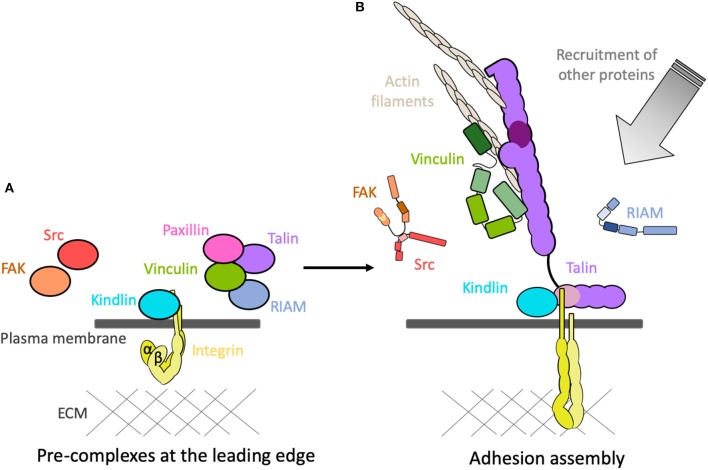
Adhesions assemble! **(A)** The coalescence of adhesion proteins into pre-complexes at the leading edge of the cell in the vicinity of the inactive integrins. **(B)** Following activation, the proteins rapidly assemble onto the cytoplasmic tails of the integrins and create mechanical linkages and signaling hubs. Other proteins are then recruited, and activated, to the adhesion site.

Many proteins in adhesion complexes form tethered linkages where the head and the tail are held apart (Figures 4F, 5B), for instance, vinculin when active forms a linkage between talin and actin. This dramatically reduces the affinity of autoinhibition as the two interacting domains are held apart and less able to interact. In these tethered systems, autoinhibition is a major mechanism enabling mechanotransduction as tethering alters the dynamics of the protein maintaining it for longer in the active state. This effect can be further enhanced if the tether is under mechanical force: if the protein is stretched, it is even harder for autoinhibition to occur (Wang et al., 2019). Many proteins can adopt multiple conformational states where binding sites are masked via intramolecular interactions and autoinhibition is where the lowest energy state results in suppression of the protein's functional role. As mechanical forces can lower the energy of active states, they can thus relieve autoinhibition.

Autoinhibition in proteins that form part of mechanical linkages in the cell are particularly sensitive to mechanical forces. Whilst force might not be required to open up talin or vinculin from their head-tail autoinhibition, force will play a major role in coordinating the lifetime of the active state (Wang et al., 2019), in essence holding the molecule in an open, extended conformation. As such, the affinity of the protein with its ligands is directly correlated to the force on the system.

## Manipulation of Autoinhibition by Mutation

Detailed biochemical characterization of the autoinhibitory domains in proteins enables the development of precise mutations that disrupt autoinhibition and render the protein constitutively active. Such mutations (examples of which are shown in Table 1) provide a way of uncoupling proteins from their activation processes and enable the detailed study of systems where the upstream signaling pathways are uncoupled. Such approaches complete a pipeline from biochemical to *in cellulo* to *in vivo* characterization and can enable detailed analysis of the protein in its active form, detached from the complex regulatory elements that control their activities.

## Case Study: E1770A—Constitutively Active Talin

The autoinhibition of talin is mediated by intramolecular and intermolecular interactions, and the major autoinhibitory domain has been mapped to the R9 of the rod domain binding to the integrin-binding site in F3.

Biochemical characterization of talin autoinhibition showed that the integrin-binding F3 domain interacted with the talin rod (Goksoy et al., 2008). Further refinement showed that the major autoinhibitory interaction was between F3 and the R9 rod domain (Goult et al., 2009), an interaction mediated by a negative surface on R9 which interacts with and occludes the basic activation loop on F3 (Wegener et al., 2007; Song et al., 2012). This structural information enabled identification of a R9 mutation (E1770A) which disrupted the interaction with F3. Analysis of this E1770A mutant in the context of full-length talin in HUVEC cells (Kopp et al., 2010) showed that a constitutively active talin leads to adhesions forming much more rapidly and in significantly larger numbers.

Introduction of the E1770A mutant in Drosophila (E1777A in fly talin) led to insight into the loss of essential developmental phenotypes (Ellis et al., 2013). In a mouse model this mutation results in various defects as a result of more mature and stable focal adhesions, which ultimately results in impeded wound healing (Haage et al., 2018).

This pipeline from *in vitro* to *in cellulo* to *in vivo*, encompassing structural and cellular methods, allows a broader understanding of autoinhibition and the consequences of disrupting such integral regulatory mechanisms. As our understanding of autoinhibition of integrin-mediated adhesion proteins develops, such a multi-disciplinary approach is likely to be an increasingly effective means of investigation.

## Pre-complexes

The mechanically-sensitive interactions that occur following integrin activation have been the subject of intensive research and are reasonably well-understood. However, how all these proteins come to be in the right place and time prior to activation is much less clear. This coalescence of proteins together prior to the assembly of force-dependent linkages would suggest the formation of pre-complexes (Figure 5A). It is likely that autoinhibition maintaining the proteins in an “off” state plays a major role in the formation of these pre-complexes. The conditions prior to formation of mechanical linkages will be very different, suggesting that some of these proteins interact in different force-independent ways. The interactions between the core proteins in the pre-complex state prior to force are likely to be fundamentally different to those that form following activation.

The precise mechanism of how these proteins interact to give rise to pre-complexes is not well-understood. Using “knock-sideways” experiments where proteins are fused with mitochondrial-targeting motifs, pre-complexes between paxillin and autoinhibited talin and vinculin have been observed (Atherton et al., 2019). Furthermore, the use of constitutively active talin and vinculin mutants targeted to mitochondria enables the study of complexes following activation of one component. Fully autoinhibited talin and vinculin do not preassemble on the mitochondria but, by simulating the tethered state of either talin or vinculin using mutation, the release of the head-tail interaction of one was sufficient to trigger interaction of the two proteins. Such technologies are a powerful way to study the most basic of questions—in a cellular context, which proteins bind which, when, and where?

The rapid development of new sophisticated microscopy techniques is allowing the observation of complexes within adhesions to be seen with unprecedented levels of detail (Kanchanawong et al., 2010), facilitating detection of multiple populations of a molecule within an adhesion. For instance, vinculin has at least three distinct states within an adhesion (Case et al., 2015) dictated by interactions with paxillin, talin and actin. Inactive vinculin is initially recruited by paxillin to the plasma membrane at the integrin adhesion “ground zero.” Subsequent vinculin activation, promoted by talin, leads to vinculin moving away from the plasma membrane and to a “signaling layer” and ultimately the “force transmission” layer.

Further, evidence for pre-complexation of inactive adhesion proteins comes from the use of fluorescent fluctuation analysis approaches (Bachir et al., 2014). Here, talin and vinculin are seen to associate before the formation of the integrin-talin complex in the nascent adhesion. More recently, new technologies utilizing machine learning trained on high-resolution traction force microscopy, coupled with single particle tracking and fluorescence fluctuation time-series analysis, is enabling the genesis of nascent adhesions to be visualized (Han et al., 2019). This visualization of the earliest stages of adhesion assembly is revealing that talin-vinculin-paxillin pre-complexes are a prerequisite for efficient and meaningful adhesion maturation. Talin and vinculin are required to be together at the moment of force generation to provide efficient maturation—if this is disrupted, maturation is limited. Nascent adhesions where talin and vinculin are recruited at different times fail to mature efficiently. Pre-complex formation appears to be required for efficient assembly of force-bearing linkages and for adhesions to mature following traction force.

It has recently been shown that many of the talin molecules at adhesion sites are non-force bearing, suggesting they are targeted to adhesion sites, ready for action, but are not all simultaneously engaged (Lemke et al., 2019). It will be interesting to explore if this is common to other adhesion molecules and what complexes maintain them at the adhesion site.

Altogether, this amounts to compelling evidence that adhesion proteins form pre-complexes prior to adhesion assembly. Having the key proteins localized together at time zero may be a requirement for rapid and productive adhesion assembly. For example, talin, vinculin and paxillin form a pre-complex (Bachir et al., 2014; Han et al., 2019) prior to association with integrins and prior to mechanical forces, and a second pre-complex of kindlin and integrin has been identified (Rossier and Giannone, 2016). Could these two pre-complexes coming together be sufficient to lead to mutual relief of autoinhibition and trigger adhesion assembly?

The use of FRET-based tension sensors (Austen et al., 2015; Kumar et al., 2016), traction force experiments, microscopy and the rapid advances in artificial intelligence, material science and microfluidics are enabling incredible insight into the interactions of proteins in mature adhesions, in 3D and nascent adhesions as the resolution improves. These technical advances are helping to answer the major open question in the field regarding how these proteins assemble and the interactions that mediate complex formation prior to activation of the integrin.

## Conclusion and Philosophical Reflections

Cells sense the chemical and mechanical properties of their environment through integrins, clustered in punctate adhesion complexes linked to the actin cytoskeleton. The precision and speed with which these adhesions assemble following activation of an integrin is remarkable and highlights the need for incredible robustness in the process of assembling such huge multiprotein complexes.

The core of most integrin adhesions comprises talin and kindlin bound to and coordinating the activation state of integrin. Once engaged to integrin, the talin rod serves as a mechanosensitive signaling hub (MSH) and, onto this hub, hundreds of proteins are recruited (Goult et al., 2018). Each protein is regulated, many by autoinhibition, and the activation status of each protein provides a regulatable point in the process. A signal leading to activation of one protein (i.e., integrin-ligand interaction outside the cell, or Rap1 activation inside the cell, etc.) might trigger a cascade of activation where autoinhibition of many proteins is simultaneously or sequentially relieved. This enables the system to exact both subtle and sweeping changes to the adhesion and signaling outputs of the adhesion.

Tight regulation and control of adhesion is crucial during development and adult tissue homeostasis. Defects in this tight regulation are implicated in many pathological conditions. Out of the 60 core consensus integrin adhesome proteins, 32 have been shown to be involved in cancer development and progression (Winograd-Katz et al., 2014). Inherited gene mutations in adhesome components are a significant source of disease and disability. Misregulation of the activity of any protein in the network will disrupt the fine balance needed to ensure the exquisitely tuned adhesive and signaling balance. Mutations in adhesion proteins and regulators give rise to diverse malfunctions and diseases as they perturb this balance in different ways which tilt the system in different tissues.

Various intra- and extracellular changes in the environment of the cell trigger subtle readjustments and changes to the adhesive structures enabling appropriate responses. The whole adhesion machinery can be affected from afar by tweaks and changes via signaling pathways that alter the regulation of adhesion proteins. Likewise, external adjustments to the world outside the cell can be propagated into the cell through these adhesions, and subtle change in the lifetime of an interaction, or small reduction in the affinity of the autoinhibition of a protein (such as by increased force extending the lifetime of a linkage), will trigger changes to the system as it reorients to this new homeostasis, adopting an altered adhesion signaling complex which augments the programming of the cell.

## Author Contributions

BG and RK wrote the manuscript.

### Conflict of Interest

The authors declare that the research was conducted in the absence of any commercial or financial relationships that could be construed as a potential conflict of interest.
